# K-carrageenan fabricated γ-irradiated graphene oxide hydrogel: A versatile anticancer nanodrug carrier system

**DOI:** 10.55730/1300-0527.3760

**Published:** 2025-05-19

**Authors:** Samina SARWAR, Sumayya AZIZ, Asif RAZA, Tariq YASIN, Nuzhat SHAFI

**Affiliations:** 1 Department of Zoology, University of Azad Jammu and Kashmir, Muzaffarabad, Pakistan; 2 Department of Chemical Engineering, Pakistan Institute of Engineering and Applied Sciences (PIEAS), Islamabad, Pakistan; 3 Department of Chemistry, Pakistan Institute of Engineering and Applied Sciences (PIEAS), Islamabad, Pakistan

**Keywords:** K-carrageenan, graphene oxide, nanodrug, hydrogel, gamma irradiatio

## Abstract

In this study, gamma-irradiated graphene oxide was incorporated into novel pH-responsive hydrogels. The biopolymer kappa carrageenan was blended with polyvinyl alcohol in a silane crosslinked biopolymer, and the effect of gamma irradiation dosage was studied. Nonhazardous tetraethoxysilane (TEOS) was used as a crosslinker. The hydrogels were characterized with chemical and thermal analysis, scanning electron microscopy, and structural analysis using a variety of analytical tools. The swelling behavior of fabricated hydrogels was assessed in various solution media. As the pH of the media increased, the swelling ratio of hydrogels decreased. All fabricated hydrogels had a high swelling ratio at acidic and neutral pH levels, with a decrease in swelling observed at basic pH. The pH-sensitive response at pH 7 make these hydrogels of potential use for controlled injectable-based drug delivery. Hydrogels with the highest swelling percentage were successfully loaded with letrozole (LTZ) to investigate the release mechanism. The drug release test was conducted in PBS and it showed that hydrogel released the LTZ in a regulated manner up to 99% in 4 h. These findings suggest that the hydrogels could serve as intelligent, responsive materials for controlled drug delivery and other biomedical applications at physiological pH.

## 1. Introduction

The use of regulated drug-delivery systems (DDSs) has increased dramatically in the last couple of decades for better regulation of drug-release kinetics in the human body. Controlling the release of drugs helps to regulate the amount of medication in the body, shield the drug from physiological conditions that would otherwise cause it to break down and be eliminated, deliver the medication precisely with the least amount of exposure, and improve patient compliance [1]. Site-specific medicine is important for treating cancer, wound dressing, and in gene therapy. Such a system is possible with biodegradable and synthetic polymers. Nevertheless, these delivery systems can be expensive, toxic, and have side effects. Thus, there is a need for such a system to be safe and cost effective, with low side effects [2]. Smart DDSs (SDDSs) can simultaneously block signal pathways, respond to stimuli, transfer drugs, and stop releasing drugs [3]. SDDSs work on the principle of transferring a required amount of medicine at a predictable time to the affected site. The controlled signals of SDDSs are not only constrained to internal conditions (e.g., pH, the concentrations of particular biomolecules, catalytic function, and redox reactions [4]) but also influenced by external factors like the light of several wavelengths, magnetic behaviors, ultrasound, and electric fields [5,6]. The basis of SDDSs can be carrier hydrogels, polymers, nanoparticles, liposomes, or nanosheets, and the smart carriers are micelles [7–9]. Hydrogels are a network of 3-dimensional chains consisting primarily of homo- or copolymers with hydrophilic characteristics. They swell without being soluble and behave as colloids in which the dispersion phase is water. The physical or chemical crosslinks give the hydrogel a specific shape and insolubility. They are highly permeable with a water content of 99%. This, combined with their soft structure and absorbance, means hydrogels can be considered a natural living tissue [10–12]. Hydrogels have various benefits as DDSs. They have good swelling properties, biocompatibility, and less toxicity depending on their composition and process handling [13]. Hydrogels are a diverse and versatile class of material that are not only of use in drug delivery but also in fabricating eye lenses, wound dressing, and in tissue manufacturing [14].

Hydrogels can be made with natural or synthetic polymers. Natural hydrogels made of collagen, fibrin, and gelatin are biocompatible, biodegradable, and biologically distinguishable moieties that help the performance of different organs. However, such polymers are mechanically weak and may have microbes or induced inflammatory immune reactions [15,16]. Various synthetic hydrogels polymers like polyvinyl alcohol (PVA), polyacrylic acid (PAA), and polyethylene oxide (PEO) are altered to produce versatile functions and degradability but lack biological characteristics of natural polymers.

Recently, the smart behavior of hydrogels combined with the flexible and tunable characteristics of materials has further increased their scope in biomaterials research. Polymer hydrogel scaffolds are effective materials for tissue engineering in the repair and regeneration of different organs and tissues. Hydrogels have been widely applied in various disciplines in biomedicine for more than 5 decades [17,18]. The incorporation of biopolymers in hydrogel fabrication facilitates the development of biodegradable, biocompatible, and nonimmunogenic drug carriers [19].

More recently, there has been more interest in fabricating hydrogels as novel DDSs. Hydrogels have the capacity for site-specific and regulated delivery of curative mediators [20]. Hydrogels have been developed as medicine carriers and discharge-rate controllers [21–25]. Biomedically synthesized hydrogels deliver drugs accurately and can release drugs in response to external stimuli. Smart polymer hydrogels adjust their position and structure according to external stimuli and have significant potential for scientific investigation and several advanced technical uses.

The natural polymer kappa carrageenan (KC) is made up of 3,6-anhydro-D-galactose monomers and D-galactose-4-sulfate in a linear sequence. There are 3 subtypes of carrageenan, but only the iota and kappa subtypes are frequently utilized for gel formation. However, the fiber structure of carrageenan has low mechanical strength [26]. Synthetic polymers can be combined with biopolymers to enhance various properties [27]. PVA is a synthetic polymer with strong mechanical properties. Hydrogels made from biopolymers blended with PVA are reliable in their physical characteristics and biocompatibility [28,29]. PVA has been used extensively by researchers for several applications in biomedicines, sorption, water treatment, and agriculture [30,31]. Despite their remarkable properties, hydrogels face limitations in drug delivery due to low loading capacity (especially for hydrophobic drugs), poor mechanical strength, low homogeneity, and inadequate stimuli response. To overcome these challenges, 2-dimensional (2D) graphene has been integrated into 3-dimensional (3D) hydrogels, enhancing their performance. This 2D–3D synergy has enabled the development of advanced graphene-based hydrogels (GBH), where graphene acts as a gelator for self-assembly or a filler for blending with various molecules, creating multifunctional GBH with improved properties.

Since the discovery of graphene in 2004, the monolayer of carbon atoms in tight arrangement in a honeycomb-like structure has drawn attention. The 2D structure of graphene has unique mechanical, thermal, physical, and electronic properties, as well as particular magnetization and a large surface area [32–35]. Graphene structures have limited chemical reactivity due to the absence of functional groups. Recently, the surface of graphene has been enhanced with oxygen, producing a derivative that is hydrophilic and contains functional groups such as hydroxyl, carbonyl, carboxyl, and epoxy [36,37]. Ionizable carboxyl groups, present in graphene oxide (GO), may exchange with metal ions and help GO disperse in some polar liquids. This improves the interfacial interaction between GO and polar molecules, which in turn increases the mechanical characteristics of polar molecules [38]. Gamma (γ) irradiation offers a safer, faster, and more efficient method for synthesizing high-purity graphene composites under ambient conditions. γ-Irradiated, graphene-based nanomaterials have applications in catalysis, energy, sensing, and biomedical fields. Carbon materials like carbon nanotubes, carbon nanofibers, and graphene enhance the photocatalytic activity of materials. Graphene has strong antibacterial effects due to its sharp edges and high surface area that allow it to penetrate bacterial membranes, causing structural damage and leakage of cellular contents. The nanosheet structure of graphene can also disrupt the integrity of cell walls, leading to bacterial cell death. Additionally, the ability of graphene to interact with microbial DNA and proteins further contributes to its antibacterial activity. The side effects of conventional cancer treatments highlight the need for less harmful alternatives, leading to the exploration of nanomaterial-based photothermal therapy (PTT), photodynamic therapy (PDT), and bioimaging. Biocompatible GO is increasingly used in hydrogels to immobilize therapeutic agents and enhance mechanical and thermal properties. GO offers therapeutic advantages, including the ability to carry bioactive molecules and improve drug-loading efficiency [39].

Letrozole (LTZ) was chosen as a model drug in this study. It is a form of aromatase inhibitor medication that is used in the treatment of breast cancer. GO-incorporated hydrogels enable controlled and sustained drug release at the tumor site, significantly improving the therapeutic efficacy of LTZ, a poorly water-soluble drug. Postsurgical hydrogel implants are a viable alternative to traditional chemotherapy because they target leftover tumor cells, reduce recurrence risks, and minimize systemic toxicity. Future research should focus on their long-term stability, in vivo efficacy, and potential translation into therapeutic applications. Injectable GO-incorporated hydrogels are a promising drug delivery platform because of their simplicity of administration, extended drug release, and enhanced bioavailability. In the current study, tetraethoxysilane (TEOS) was used as a crosslinker to create novel injectable hydrogel blends of biopolymers (KC) and synthetic polymers (PVA) with varying doses of irradiated GO. No such combination (KC, γGO, and PVA) has been reported as a DDS before. Moreover, previous DDSs were administrated orally while this study presented drug delivery through an injectable route. LTZ was loaded into the novel hydrogels to show the controlled release in PBS. The fabricated LTZ-loaded hydrogel sample released the drug in PBS in a controlled manner.

## 2. Materials and methods

### 2.1. Chemicals and material

KC was acquired from Quest International (Metro Manila, Philippines). Graphite powder was purchased from Merck (Frankfurt, Germany). PVA, potassium persulfate (KPS), vinyltriethoxysilane (VTES), hydrochloric acid (HCl), sulfuric acid (H_2_SO_4_), hydrogen peroxide (H_2_O_2_), potassium permanganate (KMnO_4_), calcium chloride (CaCl_2_.2H_2_O), sodium chloride (NaCl), and TEOS were acquired from Sigma-Aldrich (Darmstadt, Germany) and used as directed. Deionized water (DIW) was used to prepare all solutions.

### 2.2. Synthesis of GO

GO was extracted from graphite powder using a modified Marcano method [40]. The oxidation of graphite flakes was accomplished by mixing 1 g of graphite, 4 mL of orthophosphoric acid, 40 mL of sulfuric acid, and 2 g of potassium permanganate while continually stirring the mixture with a magnetic stirrer. In order to control the exothermic reaction during the addition of potassium permanganate, the whole procedure was performed in an ice bath at 4 °C. The oxidation reaction was stopped by the addition of 10 mL of hydrogen peroxide. Oxidized graphite particles (GO) were collected and rinsed with 1 M HCl and DIW. The GO particles were then incubated in a desiccator after being dried under a vacuum for 48 h.

### 2.3. Irradiation of GO

The prepared GO powder was irradiated at different irradiation doses (0 kGy, 100 kGy, and 125 kGy) using a cobalt-60 irradiator (24 kCi) at a dosage rate of 5.0 kGy/h at the Nuclear Institute of Food and Agriculture (NIFA), Peshawar, Pakistan.

### 2.4. Synthesis of the hydrogel blend

A solution casting approach was used to develop GO-loaded hydrogel blends. A measure of 0.4 g of KC powder was dissolved in 40 mL of distilled water with constant stirring at 40 °C. A measure of 0.6 g of PVA was dissolved separately in DIW at 80 °C. Irradiated GO powder (5% w/v) was dispersed in 10 mL of DIW in separate glass beakers and sonicated for 2 h. The sonicated GO solution was mixed with the KC solution at 60 °C followed by the addition of the PVA solution under continuous stirring. Then, a 4% TEOS solution was added in a dropwise manor into the KC/PVA/GO solution and further stirred for 4 h at 60 °C. The prepared solution was then transferred to a plastic container and the solution was dried room at temperature. Dried KC/PVA/GO hydrogel films were peeled off and stored in a desiccator. Formulation and identification codes of synthesized hydrogels are represented in Table 1.

### 2.5. Synthesis of the drug-loaded hydrogel blend

LTZ (30 mg) was mixed in 10 mL of methanol and added in a dropwise manor to the KC/PVA/GO solution synthesized in the previous step. After continuous stirring for 1 h, the TEOS crosslinker was gradually added at a constant temperature of 60 °C. The solution was poured into a plastic container and left to air dry.

### 2.6. Characterization of the hydrogel

#### 2.6.1. Fourier transform infrared (FT-IR) spectroscopy

Fourier transform infrared (FT-IR) spectrometry of all the hydrogel samples was carried out using a Nicolet 6700 FT-IR spectrophotometer (Thermo Fisher Scientific, Waltham, MA, USA) in the range of 4000–500 cm^−1^.

#### 2.6.2. Thermogravimetric analysis

Thermal analysis measurements were carried out using a SDTA 85 thermogravimetric analyzer (Mettler Toledo, Columbus, OH, USA) in an inert environment between 50 and 800 °C and heating rate of 20 °C/min.

#### 2.6.3. Scanning electron microscopy

Scanning electron microscopy (SEM) was used to examine the morphology of hydrogel samples using a MIRA-3 instrument (Tescan, Warrendale, PA, USA). Before scanning, the samples were freeze dried and gold coated to make their surfaces conductive before scanning.

#### 2.6.4. X-ray diffraction analysis

The crystalline structures of hydrogel samples were examined by X-Ray diffraction (XRD) using a D8 Discover diffractometer (Bruker, Billerica, MA, USA). A scan rate of 1.1/min was used to acquire the diffractograms for 2θ from 5° to 35°.

### 2.7. Adsorption studies

#### 2.7.1. Swelling behavior

The swelling behavior of developed hydrogels was investigated. Dried hydrogels (100 mg) were immersed in 30 mL DIW. Hydrogel films were weighed after regular interval. The swelling ratio of the hydrogel was calculated by the formula in Equation 1.


1 $$\text{Swelling ratio}=(\frac{{\text{W}}_{\text{f}}-{\text{W}}_{\text{i}}}{{\text{W}}_{\text{i}}})\times 100$$

Where W_f_ denotes the swelled weight at time t and W_i_ denotes the initial dry weight of samples.

### 2.8. Swelling behavior in buffer, nonbuffer, and salt solutions

Buffer and nonbuffer solutions are used to investigate the pH sensitivity of hydrogels. Buffer solutions of pH 4, 7, and 10 were obtained from Sigma-Aldrich. Nonbuffer solutions of pH 4, 7, and 10 were prepared by diluting stock solutions of NaOH (pH 14.0) and HCl (pH 1.0) with DIW. A digital pH meter was used to determine the pH of solutions. Dried hydrogel samples were placed in 30 mL of sodium chloride and calcium chloride (0.2 M to 1.0 M) solutions and their swelling behavior was assessed.

### 2.9. In vitro drug release

The in vitro drug release study of the LTZ drug-loaded hydrogels was carried out in phosphate buffer saline (PBS). Drug-loaded hydrogels were initially introduced into a container containing 100 mL of PBS at a temperature of 37 °C. Aliquots of 3 mL were periodically taken out of the solution to monitor LTZ release. To maintain a steady liquid level, 3mL of fresh PBS was redeposited.

The release of LTZ was measured using UV–vis spectrophotometry at 246 nm with a Perkin Elmer Lambda 40 UV–vis spectrophotometer (Waltham, MA, USA) with PBS serving as the reference. LTZ solutions containing 5 to 30 mg/mL of solvent were used to calculate the calibration curve. Using a calibration curve, the quantity of LTZ in the solution was determined to calculate the drug release ratio.

## 3. Result and discussion

### 3.1. FT-IR spectroscopy

**Figure 1:**
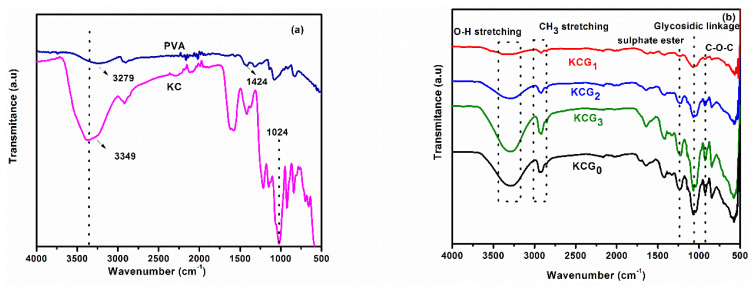
FT-IR spectra of hydrogel samples for (a) pure PVA and KC, and (b) KCG_0_, KCG_1_, KCG_2_, and KCG_3_.

FT-IR was used for chemical analysis of the samples. The obtained spectra of pure PVA, KC, KCG_0_, KCG_1_, KCG_2_, and KCG_3_ are shown in Figure 1. Observed characteristic peaks of hydrogels are summarized in Table 2. The characteristic peaks in the 3400–3000 cm^−1^ range correspond to O–H stretching vibrations. The peak at 1210–1260 cm^−1^ represents S=O stretching of sulfate groups. Peaks in the 1010–1080 cm^−1^ range are attributed to C–O–C stretching of glycosidic linkages. The characteristic peak of 3,6-anhydro-D-galactose (C–O–C stretching) is observed at 910–940 cm^−1^, while the peak at 850–840 cm^−1^ corresponds to D-galactose-4-sulfate [41]. Apart from characteristic peaks from KC, peaks characteristic of PVA were also observed in hydrogel blends at 2926 cm^−1^ in all samples of developed hydrogels (KCG_0_, KCG_1_, KCG_2_, and KCG_3_) that can be attributed to C–H alkyl stretching [42].

Comparison of FT-IR spectra of KCG_0_, KCG_1_, KCG_2_, and KCG_3_ in Figure 1b shows that all the characteristic peaks of KC, PVA, and GO are present. The O–H stretching band region (3400–3200 cm^−1^) corresponds to the hydroxyl (O–H) stretching vibrations present in both KC and PVA. In the hydrogel blend, hydrogen bonding interactions between their hydroxyl groups can lead to a broadening of this band, indicating the formation of intermolecular hydrogen bonds between the two polymers. This effect is more pronounced compared to native KC, as observed in KCG_0_.

The intensity of the peaks increases with the irradiation dose of GO. This is attributed to the presences of polar functional groups. Hydrogen bonded −OH stretching in KCG_0_, KCG_1_, KCG_2_, and KCG_3_ samples appear at 3284, 3334, 3304, and 3294 cm^−1^, respectively. Notably, the intensity of the −OH group peaks is greater in hydrogels with higher irradiation because of the abundance of polar functional groups introduced by GO [43]. The sharp bands at 1647, 1640, 1640, and 1637 cm–1 correspond to C=O stretching in KCG0, KCG1, KCG2, and KCG3 samples, respectively. The sharp band at 1647, 1640, 1640, and 1637 cm^−1^ correspond to C=O stretching. The peak intensity also increases with the irradiation dose [44]. After the incorporation of the irradiated GO into the KC/PVA composite, the stretching peak of −OH shifted to a lower wavenumber. This occurs because the irradiated GO disrupts polymer molecules via disrupting hydrogen bonding between them, and allowing the formation of new hydrogen bonds within the matrix and GO sheets. A significant increase in −OH stretching and C–O stretching is observed with higher irradiation doses [45]. The vibration in the KC biopolymer C–O–C backbone was indicated by a sharp peak at 1027 cm^−1^ and peak shifting was observed due to crosslinking of PVA and irradiated GO at 1068, 1061, 1061, and 1058 cm^−1^ in KCG0, KCG1, KCG2, and KCG3 samples, respectively. As the irradiation dose increased, the peaks shifted to a lower wavenumber. Furthermore, the peaks at 1332 and 1424 cm^−1^ confirm the existence of stretching vibrations in functional groups that contain oxygen (e.g., carboxyl, epoxy, and ester groups). The presence of all the characteristic peaks of KC, PVA, and GO in FT-IR spectra and the shifting of peaks due to the formation of hydrogen bonding with in polymer matrix confirms the KC/PVA/GO hydrogel combination was successfully synthesized [46].

### 3.2. Thermogravimetric analysis

**Figure 2:**
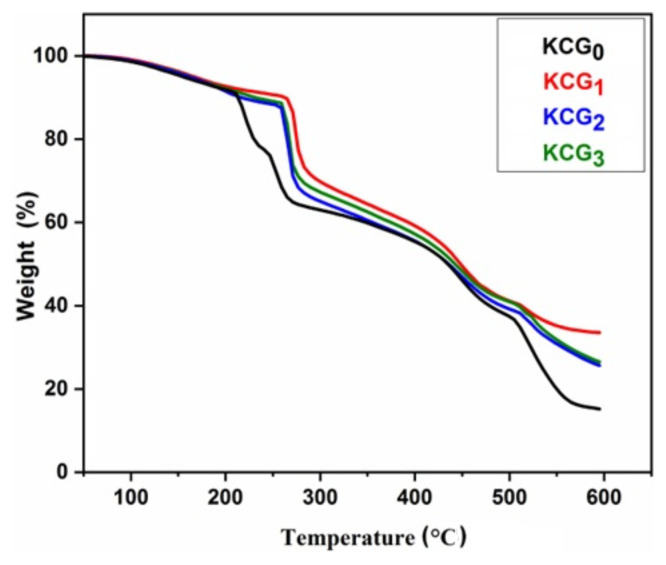
Thermograms of KC/PVA/GO hydrogel blends (KCG_0_, KCG_1_, KCG_2_, and KCG_3_).

Thermal analysis was performed to assess the thermal stability, applicability, and durability of the polymeric hydrogels. Figure 2 shows TGA thermograms of KCG_0_, KCG_1_, KCG_2_, and KCG_3_ samples. The KC/PVA/GO hydrogel has 4 thermal degradation stages at temperatures between 50–100 °C, 200–300 °C, 380–500 °C, and above 500 °C. The first degradation stage was associated with water evaporation with approximately 3.3% weight loss in all samples. The second degradation stage was associated with the disintegration of carrageenan sulfonate groups and PVA thermal degradation [47]. KCG_0_, KCG_1_, KCG_2_, and KCG_3_ samples lost 18%, 15%, 15%, and 16% weight, respectively, at 271.5 °C. This shows that hydrogel thermal stability improved due to the addition of irradiated GO. Similar results were reported by Mahdavinia et. al. [48]. The third degradation stage was associated with the degradation of the polymer backbone. Above 500 °C, KCG_0_, KCG_1_, KCG_2_, and KCG_3_ samples lost 18%, 14%, 12%, and 13%, respectively due to the breakdown of oxygen functional groups. This shows that the stability of hydrogels increased with the addition of irradiated GO up to 100 kGy. However, a further increase in irradiation dose up to 125 kGy decreases the thermal stability of the hydrogel, possibly due to the formation of defects in GO.

Table 3 shows temperatures at 20%, 50%, and 70% mass losses along with residual mass loss of each hydrogel. Analysis of degradation temperatures and residual mass shows that KCG_2_ is more thermally stable.

### 3.3. X-ray diffraction analysis

**Figure 3:**
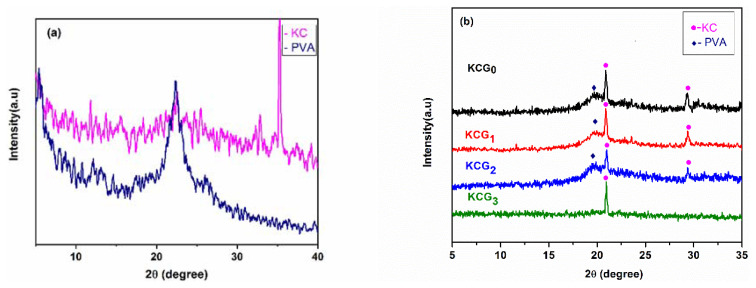
XRD diffractograms for (a) pure KC and PVA, and (b) hydrogel blends KCG_0_, KCG_1_, KCG_2_, and KCG_3_.

XRD is used to determine the structural characteristics, crystalline orientation, and crystalline phases in polymers. The XRD diffractogram of pure PVA, pure KC, and hydrogel samples are shown in Figure 3. Distinctive peaks corresponding to PVA are shown at 2θ = 19.9° in Figure 3a. In the diffractogram for KC, there is a weak broad peak near 2θ ≈ 20° in Figure 3a, indicating its amorphous nature [49]. The XRD pattern of GO shows an intense and sharp peak centered at 10.24° [50].

Figure 3b shows the XRD results of KCG_0_, KCG_1_, KCG_2_, and KCG_3_ hydrogel samples. Sharp diffraction peaks are observed at 2θ = 20.6° and 28° in all samples. A decrease in intensity isobserved with the increase in irradiation dose due to increased intermolecular interactions between the KC, PVA, and GO molecules. Furthermore, when GO was mixed with the PVA/KC solution, the hydrogel only displays the KC/PVA diffraction peaks; the distinctive GO peak is not present. This might be the result of the relatively low concentration of GO in the PVA/KC solution and the fact that the diffraction of GO is much weaker than that of KC/PVA [51].

The positions of the diffraction peaks shift slightly after the addition of GO to the hydrogel. Furthermore, the intensities of these peaks decreased considerably in contrast to the nonirradiated composite sample. The peak intensity decreased gradually as the irradiation dose increased. Increased molecular interaction due to the addition of irradiated GO causes a significant decrease in the degree of crystallinity of the hydrogel blends (KCG_1_, KCG_2_, and KCG_3_).

### 3.4. Scanning electron microscopy

**Figure 4:**
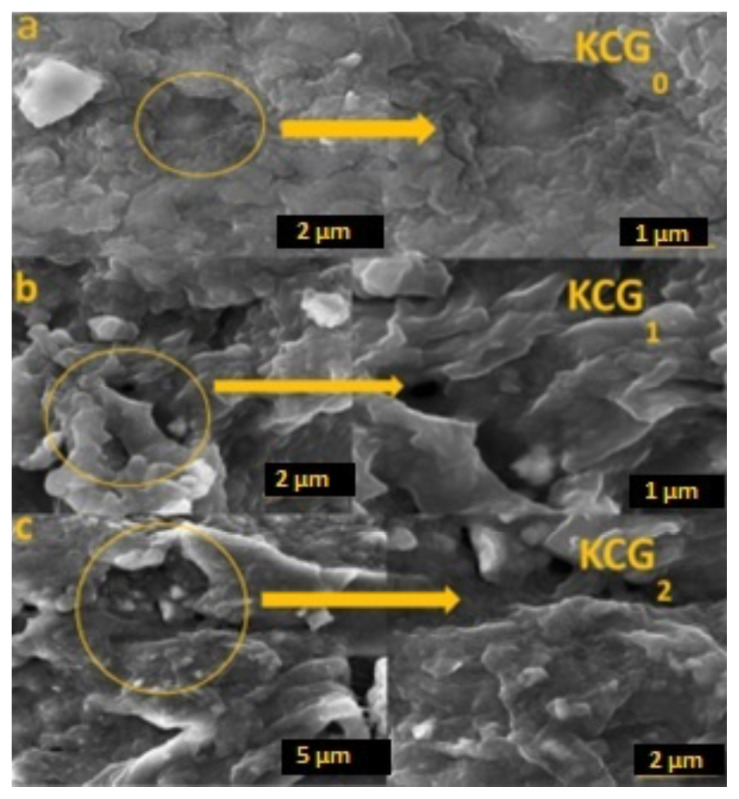
SEM Images of (a) KCG_0_, (b) KCG_1_, and (c) KCG_2_

SEM was used to examine the morphology of freeze-dried hydrogels. Representative SEM micrographs of hydrogels without GO (KCG_0_), with nonirradiated GO (KCG_1_), and GO irradiated to 100 kGy (KCG_2_) are shown in Figure 4. KCG_0_ had a smooth surface (Figure 4a), whereas hydrogels with GO were coarse with the appearance of wrinkles (Figure 4b and 4c). Additionally, the surface morphology of the composite hydrogels showed large holes that aided in the diffusion of molecules in the adsorption tests. The smaller, denser pores and wrinkles provide a larger specific surface that enhanced the absorption ability of the hydrogel. KCG_3_ did not show satisfactory results in swelling behavior, so SEM images are excluded. With the increase in surface roughness, hydrogel swelling increased due to increased water uptake, reduced water diffusion barriers, and increased surface area availability for water interaction.

### 3.5. Mechanism of hydrogel synthesis and drug release

Scheme illustrates how silane crosslinkers, PVA, and KC have both chemical and physical electrostatic attractions that contribute to the stable structure of the hydrogel. Additionally, these interactions make the hydrogel suitable for injection, highlighting its potential as an optimal carrier for drug delivery applications.

### 3.6. Swelling tests in various swelling media

#### 3.6.1. Swelling kinetics

The solvent distribution from the extracellular matrix to the hydrogel determines how much swelling will occur. Equation 2 was used to validate the swelling data [52].


2 F=kt n

Where k is the rate of swelling constant and F stands for fractional swelling that can be described as in Equation 3:


3 $$\text{F}=\frac{{\text{W}}_{\text{eq}}}{{\text{W}}_{\text{t}}}$$

**Figure 5:**
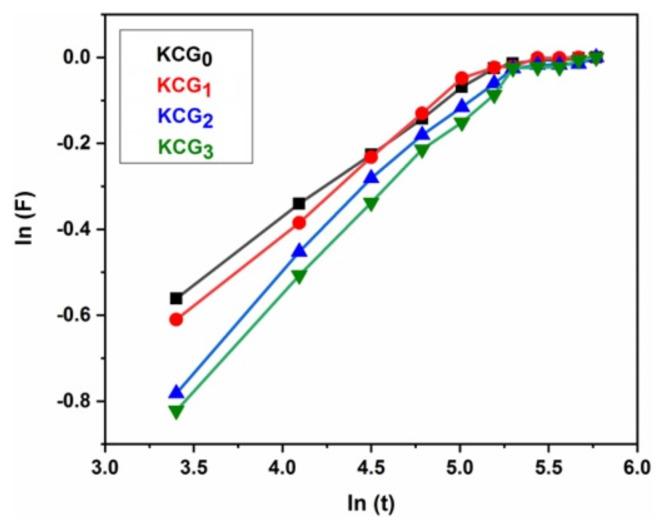
ln (F) vs ln (t) for KCG_0_, KCG_1_, KCG_2_, and KCG_3_ hydrogels.

The swelling at equilibrium time is denoted by W_eq_ and the swelling at time t by W_t_. The swelling properties of hydrogels in distilled water were used to calculate the values of k and n, where the solvent transport mechanism inside the hydrogels is defined by the value of n. When the value of n is less than or equal to 0.5, it indicates Fickian transport and a value between 0.5 and 1 indicates non-Fickian transport. Using the swelling values of hydrogels in water, a graph was plotted between ln t and ln F as illustrated in Figure 5. Table 4 presents the calculated values for the diffusion parameters, showing that the hydrogels have Fickian diffusion mechanisms.

#### 3.6.2. Swelling study of hydrogels

**Figure 6:**
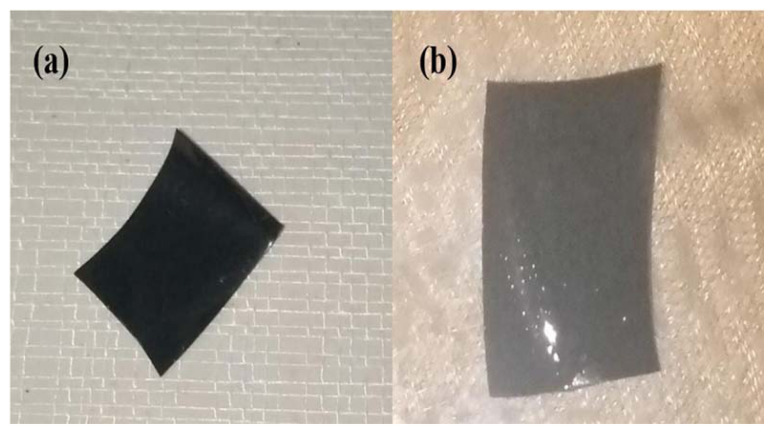
Real images of hydrogel KCG_2_ (a) before and (b) after swelling.

The ability to swell is a characteristic feature of hydrogels. Hydrogels are polymeric networks with the capacity to retain large amounts of water in various percentages depending on their chemical composition. Both chemical composition and swelling media play significant roles in the swelling behavior of hydrogels. The degree to which ionic polymers swell depends on various structural parameters, includes hydrophilicity, crosslinked density, charge, concentration, and degree of ionization. Other factors that affect the degree of swelling include the valency, pH, ionic strength, and counter ions in the swelling medium [53]. A preliminary investigation into the swelling behavior of hydrogels was conducted and their dynamic swelling behaviors in distilled water are illustrated in Figure 6. Initially, water uptake in hydrogels rapidly increased and subsequently plateaued as equilibrium was reached. This trend aligns with typical hydrogel swelling behavior, where an initial swift absorption phase is followed by a slower approach to equilibrium. The swelling behavior of the hydrogel in saline solutions differed noticeably from that seen in deionized water and was influenced by the kind of salts utilized. In order to assess the impact of pH on hydrogel swelling, samples were studied at pH 4, 7, and 10.

#### 3.6.3. Swelling ratio of hydrogel in distilled water

**Figure 7:**
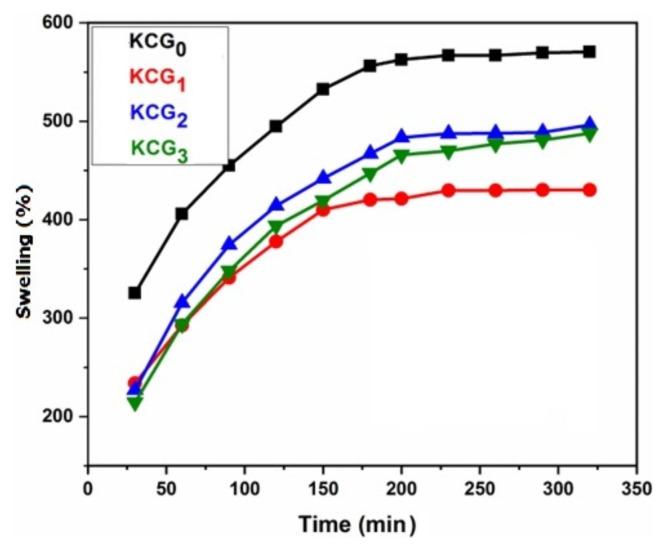
Swelling ratio of KC/PVA/GO hydrogels KCG_0_, KCG_1_, KCG_2_, and KCG_3_.

Hydrogels use diffusion to effectively absorb water. The attraction between the polymer chains and the surrounding media leads to diffusion. Figure 7 shows the swelling behavior of KC/PVA/GO hydrogels in distilled water. The swelling behavior of the hydrogel films vary widely. The stability time was 5 h for all hydrogel samples. The gel samples were placed in distilled water at room temperature and immersed until the gel reached its equilibrium condition of swelling. The hydrogel was removed from the medium, the extra solvent was quickly blotted off the surface with absorbent paper, and the sample was weighed and the average of 3 readings were reported. Figure 7 clearly shows the rate of swelling and water absorption are influenced by the composition of the hydrogels. All hydrogels containing GO had a decreased swelling capacity than hydrogels without GO added. The inclusion of GO nanoparticles is responsible for the decrease in water absorbency. Due to the presence of numerous functional groups in GO, the interaction between nanoparticles and polymeric chains results in increased crosslinking density, thereby decreasing swelling capacity [54,55].

Swelling of the hydrogel blends can be attributed to deprotonation of sulfate groups (−OSO_3_H) of carrageenan at pH 7, resulting in the creation of sulfate ions (−OSO_3_). Due to electrostatic repulsion caused by negatively charged sulfate ions repelling the sulfate ions on another chain, polymeric chains expand and lead to an increase in swelling [56]. The presence of the hydrophilic (−OH) group in PVA also had an impact on the swelling behavior [57].

#### 3.6.4. Effect of irradiation dose of GO

The effect of gamma irradiation on hydrogels was calculated by its swelling behavior. The swelling curves of hydrogels containing GO samples irradiated at different doses (0, 100, and 125 kGy) are shown in Figure 6. At first, swelling increased with the addition of GO irradiated to 100 kGy. This is because of more hydrophilic groups in GO by irradiation. As more hydrophilic hydroxyl and carboxyl functional groups are available, they are more likely to interact with water molecules. However, samples containing GO irradiated at 125 kGy showed a decrease in swelling. This may be caused by the aggregation of GO and an increase in crosslinking density. This produces hydrogels with a more compact structure due to the ability of irradiated GO to trigger crosslinking reactions [58].

#### 3.6.5. Swelling in buffer solution

**Figure 8:**
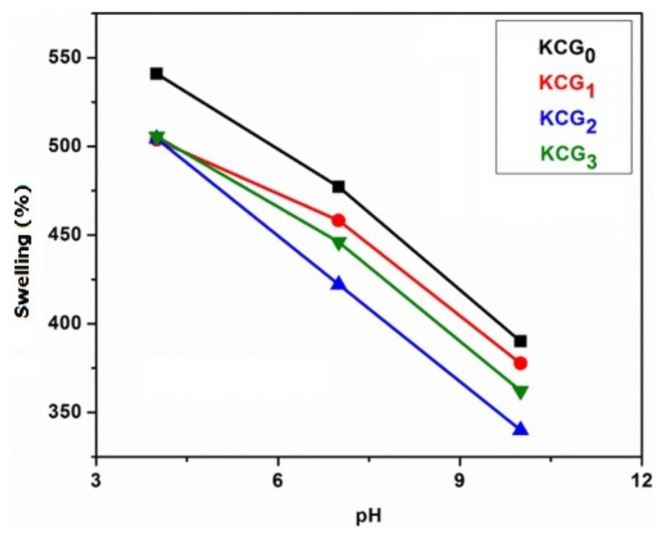
Effect of buffer solution pH on swelling of KC/PVA/GO hydrogels KCG_0_, KCG_1_, KCG_2_, and KCG_3_.

The response of prepared hydrogels to buffers with pH values of 4, 7, and 10 was examined. Figure 8 shows the effect of buffer solution pH on swelling behavior of hydrogels. At acidic pH, all samples showed the maximum swelling, moderate swelling was seen at pH 7, and decreased swelling was noted at basic pH. The protonation of the OH group of PVA and −SO_3_ of the KC caused the swelling in acidic media. Higher concentrations of charged units and ionic groups in an acidic medium cause charge repulsion that raises the swelling ratio when more solvent enters by osmosis. All hydrogels, on the other hand, consistently declined in swelling as the pH change from acidic to basic, with minimal swelling in basic conditions because of a decrease in the ionization of charged units. This reduction results in the contraction of polymer chains, thereby decreasing the swelling capacity. Maximum swelling was observed by KCG_0_ among all the samples.

#### 3.6.6. Swelling in nonbuffer

**Figure 9:**
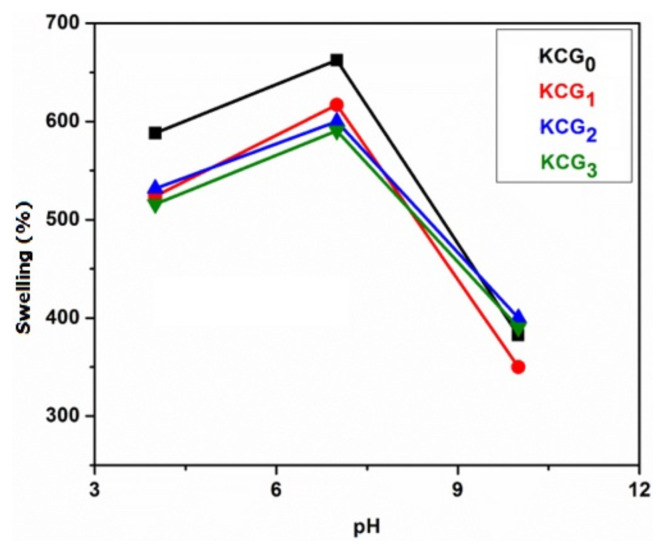
Effect of nonbuffer solution pH on swelling of KC/PVA/GO hydrogels KCG_0_, KCG_1_ KCG_2_, and KCG_3_.

The swelling response of the hydrogels in nonbuffer solutions at pH values of 4, 7, and 10 is shown in Figure 9. Swelling of hydrogels increased with pH 4 to pH 7 due to ionization of the sulphonic acid groups, i.e. OSO_3_H that imparts negative charge to the KC back bone. Free ion concentration within the gel phase increases due to ionization. As a result, it causes an increase in swelling. All hydrogel blends showed a consistent decline in swelling from an acidic to a basic pH, due to the reduced ionization of charged units, causing polymer chain constriction and a subsequent reduction in swelling across all hydrogels [59].

#### 3.6.7. Swelling in electrolytes

**Figure 10:**
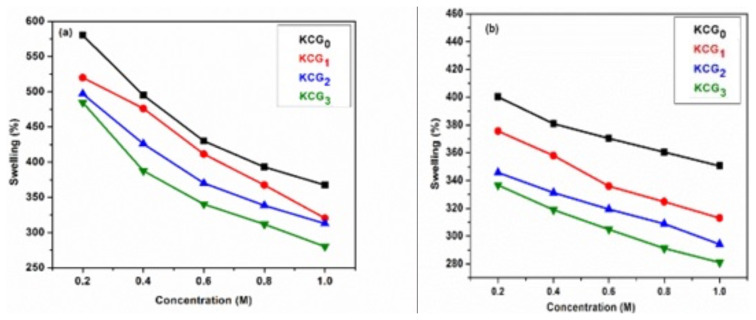
Effect of electrolyte solution (a) NaCl and (b) CaCl_2_ on swelling of KC/PVA/GO hydrogels KCG_0_, KCG_1_, KCG_2_, and KCG_3_

The effect of NaCl and CaCl_2_ concentration (0.2, 0.4, 0.6, 0.8, and 1 M) on adsorption was also studied and results are presented in Figure 10. NaCl is a monovalent Na^+^ salt and CaCl_2_ is a divalent Ca^+2^ salt. Figure 10a shows the effect of NaCl concentrations on swelling of hydrogels. It is evident that swelling percentage decreases with the increase in NaCl concentration from 0.2 to 1 M. The adsorption capacity of hydrogels was slightly reduced with the addition of sodium chloride compared to distilled water. As the polarity of the solution increased, hydrogel swelling capacity decreased. The neutralization of anionic sulfate (−OSO_3_) groups causes the reduction in swelling of hydrogels [60,61].

The adsorption capability of hydrogels in the CaCl_2_ solutions was noticeably lower than in the NaCl solution, as shown in the Figure 10b. This is caused by Ca ion interaction with hydrogel anionic centers. The screening effect of excessive charge present in the electrolyte solution causes a decrease in swelling ratio. These compounds can cause hydrogels to contract and, as a result, have a smaller surface area. Adsorption capability of the hydrogels decreases as surface area reduced. An increase in the molar concentration of the electrolytes also causes a decrease in the osmotic pressure between the electrolytes and the hydrogel matrix, which prevents water from entering into the hydrogel matrix [62].

### 3.7. Controlled release examination of LTZ

**Figure 11:**
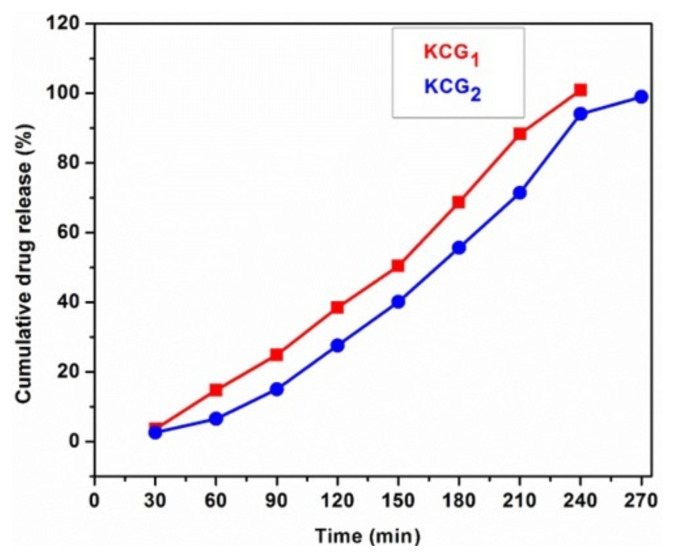
Release study of LTZ in PBS solution over time.

In vitro studies were carried out to study the drug release activity due to the fact that the hydrogels swell more in acidic pH, thus limiting their use for oral drug delivery. The drug release behavior of the hydrogels is closely associated with their swelling properties and a crucial aspect of hydrogel structural design. As the hydrogel swells, its mesh size expands, creating diffusion pathways that facilitate drug release. LTZ was selected as the model drug, and loaded on KCG_1_ and KCG_2_ hydrogels. KCG_3_ was not used for drug loading due low swelling. The release mechanism of these hydrogels was then examined in PBS with respect to time (t) at 37 °C. (Figure 11). The hydrogels KCG_1_ and KCG_2_ both showed sustained release of LTZ over a period of 5 h. Higher irradiation of GO (KCG_2_) caused slower drug release. In PBS, LTZ was released slowly; 99% of the whole dose was released in 4.5 h compared to 100% in 4 h by KCG_1_. Slow release may be attributed to the addition of GO, as GO has a large surface area with abundant functional groups (e.g., carboxyl, hydroxyl, and epoxy) that enable strong hydrogen bonds and π–π interactions with drug molecules. These interactions resulted in higher drug retention within the hydrogel matrix, decreasing its release. LTZ, being a poorly water-soluble drug, may have strong hydrophobic interactions with GO, further reducing its release in an aqueous environment like PBS. Therefore, the higher controlled release behavior of KCG_2_ highlight its potential for intravenous medications.

## Conclusion

KC/PVA hydrogels combined with various doses of irradiated GO were developed and their chemical, morphological, thermal, swelling, and drug delivery properties were characterized. The hydrogel showed increased thermal stability, swelling behavior, and injection ability properties as irradiation dose increased. Water-retention capacity increased when GO was incorporated. KCG_2_ (100 kGy) showed maximum swelling and thermal stability. This increase in swelling facilitated a more efficient drug diffusion process by expanding the hydrogel network. The drug release from the hydrogels was primarily governed by a swelling-controlled diffusion mechanism. The presence of GO contributed to a more sustained and controlled release profile, reducing the initial burst release commonly seen in traditional hydrogels. The Fickian diffusion regulation mechanism was also studied in all hydrogels and the value of diffusional exponent n was found to vary from 0.243 in KCG_0_ to 0.068 in KCG_3_. The pH swelling response of hydrogels indicated high swelling ratio in acidic conditions and low swelling ratio in basic conditions. Maximum swelling was observed at around neutral pH, making these complexes particularly appropriate candidate for injectable DDSs. In drug loading and release investigations, KCG_1_ and KCG_2_ hydrogels were tested. LTZ was released in a sustained manner for a period of 4 h. This research highlights the significance of GO-based hydrogels for biomedical applications including drug delivery, tissue engineering, and wound healing.

## Figures and Tables

**Scheme:**
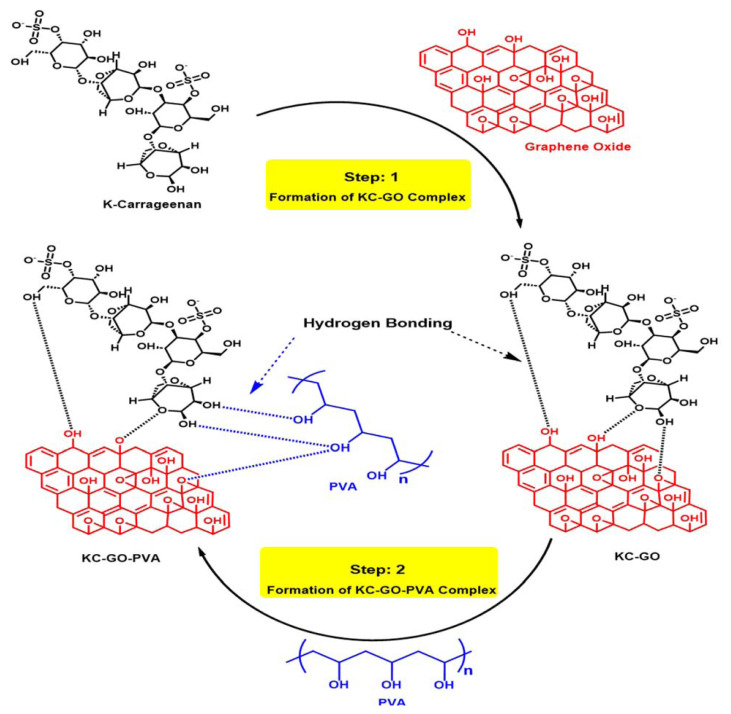
Loading mechanism of LTZ drug in KC/PVA/GO hydrogel blend.

**Table 1:** Formulation and identification codes of synthesized hydrogels.

Sample code	KCG0	KCG_1_	KCG_2_	KCG_3_
GO kGy	No graphene	0 kGy	100 kGy	125 kGy

PVA = 60%, KC = 40 %, TEOS = 4%

**Table 2:** Summary of characteristic peaks in FT-IR spectra of KCG_0_, KCG_1_, KCG_2_, and KCG_3_.

Functional groups	Absorbance (cm^−1^) range	Observed Absorbance (cm^−1^)			

		KCG_0_	KCG_1_	KCG_2_	KCG_3_
O-H stretching	3200–3400	3284	3334	3304	3294
Glycosidic linkage	1010–1080	1068	1061	1061	1058
Sulfate easter	1210–1260	1236	1232	1225	1225
3,6-anhydro-D-gactose	910–940	919	919	919	919
D-galactose-4-sulfate	840–850	844	859	841	836
CH_3_ asymmetrical stretching vibrations	2927	2926	2926	2926	2926
C=O stretching	1648	1647	1640	1640	1637

**Table 3:** Thermal degradation of hydrogel blends at different weight loss rates.

Sample	T_20%_ (ºC)	T_50%_ (ºC)	T_70%_(ºC)	Residue %
KCG_0_	232.01	434.03	525.0	15.26
KCG_1_	264.0	433.11	549.2	25.63
KCG_2_	274.27	447.10	586.72	33.53
KCG_3_	267.85	444.04.	560.96	26.51

**Table 4:** Diffusion parameters of KCG_0_, KCG_1_, KCG_2_, and KCG_3_.

Parameters	KCG_0_	KCG_1_	KCG_2_	KCG_3_
N	0.243	0.2658	0.326	0.0688
Intercept	−1.33561	−1.45422	−1.8007	−1.9524
K	0.243041	0.265775	0.326383	0.35235

## References

[1] Adepu S Ramakrishna S Controlled drug delivery systems: current status and future directions Molecules 2021 26 19 5905 10.3390/molecules26195905 34641447 PMC8512302

[2] Sung YK Kim SW Recent advances in polymeric drug delivery systems Biomaterials Research 2020 24 1 12 10.34133/bmr.0221 32537239 PMC7285724

[3] Rana A Adhikary M Singh PK Das BC Bhatnagar S “Smart” drug delivery: A window to future of translational medicine” Frontiers in Chemistry 2023 10 1095598 10.3389/fchem.2022.1095598 36688039 PMC9846181

[4] Zhang H Fan T Chen W Li Y Wang B Recent advances of two-dimensional materials in smart drug delivery nano-systems Bioactive Materials 2020 5 4 1071 1086 10.1016/j.bioactmat.2020.06.012 32695937 PMC7363990

[5] Xiong F Huang S Gu N Magnetic nanoparticles: recent developments in drug delivery system Drug Development and Industrial Pharmacy 2018 44 5 697 706 10.1080/03639045.2017.1421961 29370711

[6] Kolosnjaj-Tabi J Gibot L Fourquaux I Golzio M Rols MP Electric field-responsive nanoparticles and electric fields: physical, chemical, biological mechanisms and therapeutic prospects Advanced Drug Delivery Reviews 2019 138 56 67 10.1016/j.addr.2018.10.017 30414494

[7] Hossen S Hossain MK Basher MK Mia MNH Rahman MT Smart nanocarrier-based drug delivery systems for cancer therapy and toxicity studies: A review Journal of Advanced Research 2019 15 1 18 10.1016/j.jare.2018.06.005 30581608 PMC6300464

[8] Crossen SL Goswami T Nanoparticulate carriers for drug delivery Journal of Pharmaceutical and Biopharmaceutical Research 2022 4 1 237 10.25082/JPBR.2022.01.001

[9] Lombardo D Kiselev MA Caccamo MT Smart nanoparticles for drug delivery application: development of versatile nanocarrier platforms in biotechnology and nanomedicine Journal of Nanomaterials 2019 1 3702518 10.1155/2019/3702518

[10] Das S Kumar V Tiwari R Singh L Singh S Recent advances in hydrogels for biomedical applications Asian Journal Pharmaceutical Clinical Research 2018 11 11 62 68 10.22159/ajpcr.2018.v11i11.27921

[11] Lukatsky ATD Dan Y Mizrahi L Amir E Hydrogels based on crosslinked polyethylene glycol diacrylate and fish skin gelatin European Polymer Journal 2024 210 112990 10.1016/j.eurpolymj.2024.112990

[12] Omidian H Akhzarmehr A Chowdhury SD Advancements in cellulose-based superabsorbent hydrogels: Sustainable solutions across industries Gels 2024 10 3 174 10.3390/gels10030174 38534592 PMC10970592

[13] Ghasemiyeh P Mohammadi-Samani S Hydrogels as drug delivery systems; pros and cons Trends in Pharmaceutical Sciences 2019 5 1 7 24 10.30476/TIPS.2019.81604.1002

[14] Kesharwani P Bisht A Alexander A Dave V Sharma S Biomedical applications of hydrogels in drug delivery system: An update Journal of Drug Delivery Science and Technology 2021 66 102914 10.1016/j.jddst.2021.102914

[15] Akhtar S Singh G Singh SP Kumar S Malik JK Current Scenario of Hydrogel as Drug Delivery System South Asian Research Journal Pharmaceutical Sciences 2023 5 2 33 37 10.36346/sarjps.2023.v05i02.002

[16] Shoukat H Buksh K Noreen S Pervaiz F Maqbool I Hydrogels as potential drug-delivery systems: Network design and applications Therapeutic Delivery 2021 12 5 375 396 10.4155/tde-2020-0114 33792360

[17] Revete A Aparicio A Cisterna BA Revete J Luis L Advancements in the use of hydrogels for regenerative medicine: properties and biomedical applications International Journal of Biomaterials 2022 1 3606765 10.1155/2022/3606765 PMC9663251 36387956

[18] Sánchez-Cid P Jiménez-Rosado M Romero A Pérez-Puyana V Novel trends in hydrogel development for biomedical applications: A review Polymers 2022 14 15 3023 10.3390/polym14153023 35893984 PMC9370620

[19] Saharan R Paliwal SK Tiwari A Babu MA Tiwari V Beyond traditional hydrogels: The emergence of graphene oxide-based hydrogels in drug delivery Journal of Drug Delivery Science and Technology 2024 105506 10.1016/j.jddst.2024.105506

[20] Ezike TC Okpala US Onoja UL Nwike CP Ezeako EC Advances in drug delivery systems, challenges and future directions Heliyon 2023 9 6 10.1016/j.heliyon.2023.e17488 PMC10320272 37416680

[21] Narayanaswamy R Torchilin VP Hydrogels and their applications in targeted drug delivery The Road from Nanomedicine to Precision Medicine 1st Edition Jenny Stanford Publishing 2019 34 10.3390/molecules24030603 PMC6384686 30744011

[22] Raina N Pahwa R Bhattacharya J Paul AK Nissapatorn V Drug delivery strategies and biomedical significance of hydrogels: translational considerations Pharmaceutics 2022 14 3 574 10.3390/pharmaceutics14030574 35335950 PMC8950534

[23] Majumder J Minko T Multifunctional and stimuli-responsive nanocarriers for targeted therapeutic delivery Expert Opinion on Drug Delivery 2021 18 2 205 227 10.1080/17425247.2021.1828339 32969740 PMC7904578

[24] Gomte SS Agnihotri TG Khopade S Jain A Exploring the potential of pH-sensitive polymers in targeted drug delivery Journal of Biomaterials Science, Polymer 2024 35 2 228 268 10.1080/09205063.2023.2279792 37927045

[25] Chyzy A Tomczykowa M Plonska-Brzezinska ME Hydrogels as potential nano-, micro-and macro-scale systems for controlled drug delivery Materials 2020 13 1 188 10.3390/ma13010188 31906527 PMC6981598

[26] Rasool A Ata S Islam A Khan RU Fabrication of novel carrageenan based stimuli responsive injectable hydrogels for controlled release of cephradine RSC Advances 2019 9 22 12282 12290 10.1039/C9RA02130B 35515844 PMC9063494

[27] Gobi R Ravichandiran P Babu RS Yoo DJ Biopolymer and synthetic polymer-based nanocomposites in wound dressing applications: a review Polymers 2021 13 12 1962 10.3390/polym13121962 34199209 PMC8232021

[28] Feldman D Poly (vinyl alcohol) recent contributions to engineering and medicine Journal of Composites Science 2020 4 4 175 10.3390/jcs4040175

[29] Sun X Luo C Luo F Preparation and properties of self-healable and conductive PVA-agar hydrogel with ultra-high mechanical strength European Polymer Journal 2020 124 109465 10.1016/j.eurpolymj.2019.109465

[30] Abdlhammed AF Abbas MAK Sahab QM Ibrahim MBM Review article about polyvinyl alcohol reactions European Journal of Modern Medicine and Practice 2024 4 8 239 253 10.1016/j.radphyschem.2024.112269

[31] Zhang K Liu Y Shi X Zhang R He Y Application of polyvinyl alcohol/chitosan copolymer hydrogels in biomedicine: A review International Journal of Biological Macromolecules 2023 242 125192 10.1016/j.ijbiomac.2023.125192 37276897

[32] Farjadian F Abbaspour S Sadatlu MAA Mirkiani S Ghasemi A Recent developments in graphene and graphene oxide: Properties, synthesis, and modifications: A review Chemistry Select 2020 5 33 10200 10219 10.1002/slct.202002501

[33] Mushahary N Sarkar A Basumatary F Brahma S Das B Recent developments on graphene oxide and its composite materials: From fundamentals to applications in biodiesel synthesis, adsorption, photocatalysis, supercapacitors, sensors and antimicrobial activity Results in Surfaces and Interfaces 2024 100225 10.1016/j.rsurfi.2024.100225

[34] Catania F Marras E Giorcelli M Jagdale P Lavagna L A review on recent advancements of graphene and graphene-related materials in biological applications Applied Sciences 2021 11 2 614 10.3390/app11020614

[35] Anegbe B Ifijen IH Maliki M Uwidia IE Aigbodion AI Graphene oxide synthesis and applications in emerging contaminant removal: a comprehensive review Environmental Sciences Europe 2024 36 1 15 10.1186/s12302-023-00814-4

[36] Khine YY Wen X Jin X Foller T Joshi R Functional groups in graphene oxide Physical Chemistry Chemical Physics 2022 24 43 26337 26355 10.1039/D2CP04082D 36285559

[37] Magne TM de Oliveira VT Alencar LMR Junior FFM Gemini-Piperni S Graphene and its derivatives: understanding the main chemical and medicinal chemistry roles for biomedical applications Journal of Nanostructure in Chemistry 2022 1 35 10.1007/s40097-021-00444-3 34512930 PMC8419677

[38] He Y Liu Y Guo F Pang K Fang B Dynamic dispersion stability of graphene oxide with metal ions Chinese Chemical Letters 2020 31 6 1625 1629 10.1016/j.cclet.2019.10.010

[39] Zikalala NE Azizi S Thema FT Cloete KJ Zinatizadeh AA Modification of graphene-based nanomaterials with gamma irradiation as an eco-friendly approach for diverse applications: A review Flat Chem 2024 100662 10.1016/j.flatc.2024.100662

[40] Husnah M Fakhri HA Rohman F Aimon AH Iskandar F A modified Marcano method for improving electrical properties of reduced graphene oxide (rGO) Materials Research Express 2017 4 6 064001 10.1088/2053-1591/aa707f

[41] Liew JWY Loh KS Ahmad A Lim KL Wan Daud WR Synthesis and characterization of modified κ-carrageenan for enhanced proton conductivity as polymer electrolyte membrane Plos one 2017 12 9 e0185313 10.1371/journal.pone.0185313 28957374 PMC5619736

[42] Bajpai SK Daheriya P Kappa-carrageenan/PVA films with antibacterial properties: Part 1. Optimization of preparation conditions and preliminary drug release studies Journal of Macromolecular Science 2014 51 4 286 295 10.1080/10601325.2014.882687

[43] Yadav S Ibrar I Altaee A Samal AK Zhou J Surface modification of nanofiltration membrane with kappa-carrageenan/graphene oxide for leachate wastewater treatment Journal of Membrane Science 2022 659 120776 10.1016/j.memsci.2022.120776

[44] Zakaria Z Kamarudin SK Kudus MHA Wahid KAA K-carrageenan/polyvinyl alcohol-graphene oxide biopolymer composite membrane for application of air-breathing passive direct ethanol fuel cells Journal of Applied Polymer Science 2022 139 22 52256 10.1002/app.52256

[45] Shi Y Xiong D Li J Wang K Wang N In situ repair of graphene defects and enhancement of its reinforcement effect in polyvinyl alcohol hydrogels Rsc Advances 2017 7 2 1045 1055 10.1039/C6RA24949C

[46] Meng F Zhang Y Xiong Z Wang G Li F Mechanical, hydrophobic and thermal properties of an organic-inorganic hybrid carrageenan-polyvinyl alcohol composite film Composites Part B: Engineering 2018 143 1 8 10.1016/j.compositesb.2017.12.009

[47] Gad YH Nasef SM Radiation synthesis of graphene oxide/composite hydrogels and their ability for potential dye adsorption from wastewater Journal of Applied Polymer Science 2021 138 41 51220 10.1002/app.51220

[48] Mahdavinia GR Etemadi H In situ synthesis of magnetic CARAPVA IPN nanocomposite hydrogels and controlled drug release Materials Science and Engineering 2014 45 250 260 10.1016/j.msec.2014.09.023 25491827

[49] Chandika P Kim M Khan F Kim Y Heo S Wound healing properties of triple cross-linked poly (vinyl alcohol)/methacrylate kappa-carrageenan/chitooligosaccharide hydrogel Carbohydrate Polymers 2021 269 118272 10.1016/j.carbpol.2021.118272 34294304

[50] Vinothini K Rajendran NK Munusamy MA Alarfaj AA Rajan M Development of biotin molecule targeted cancer cell drug delivery of doxorubicin loaded κ-carrageenan grafted graphene oxide nanocarrier Materials Science and Engineering 2019 100 676 687 10.1016/j.msec.2019.03.011 30948104

[51] Shuai C Feng P Gao C Shuai X Xiao T Graphene oxide reinforced poly (vinyl alcohol): Nanocomposite scaffolds for tissue engineering applications RSC Advances 2015 5 32 25416 25423 10.1039/C4RA16702C

[52] Farooq A Farooq A Jabeen S Islam A Gull N Designing Kappa-carrageenan/guar gum/polyvinyl alcohol-based pH-responsive silane-crosslinked hydrogels for controlled release of cephradine Journal of Drug Delivery Science and Technology 2022 67 102969 10.1016/j.jddst.2021.102969

[53] Mahdavinia GR Etemadi H Soleymani F Magnetic/pH-responsive Beads Based on Caboxymethyl Chitosan and K-carrageenan and Controlled Drug Release Carbohydrate Polymers 2015 128 112 121 10.1016/j.carbpol.2015.04.022 26005146

[54] Ma T Chang PR Zheng P Zhao F Ma X Fabrication of ultra-light graphene-based gels and their adsorption of methylene blue Chemical Engineering Journal 2014 240 595 600 10.1016/j.cej.2013.10.077

[55] Rather RA Bhat MA Shalla AH Multicomponent interpenetrating metal based Alginate-Carrageenan biopolymer hydrogel beads substantiated by graphene oxide for efficient removal of methylene blue from waste water Chemical Engineering Research and Design 2022 182 604 615 10.1016/j.cherd.2022.04.017

[56] Distantina S Rochmadi R Fahrurrozi M Wiratni W Preparation and characterization of glutaraldehyde-crosslinked kappa carrageenan hydrogel Engineering Journal 2013 17 3 57 66

[57] Khaliq T Sohail M Minhas MU Shah SA Jabeen N Self-crosslinked chitosan/κ-carrageenan-based biomimetic membranes to combat diabetic burn wound infections International Journal of Biological Macromolecules 2022 197 157 168 10.1016/j.ijbiomac.2021.12.100 34968540

[58] Islam MT Dafader NC Poddar P Khan MS Chowdhury AM Studies on swelling and absorption properties of the γ–irradiated polyvinyl alcohol (PVA)/kappa-carrageenan blend hydrogels Radiation Physics and Chemistry 2016 122 1 8 10.1016/j.radphyschem.2022.110473

[59] Islam A Yasin T Bano I Riaz M Controlled release of aspirin from pH-sensitive chitosan/poly (vinyl alcohol) hydrogel Journal of Applied Polymer Science 2012 124 5 4184 4192 10.1002/app.35392

[60] Li Y Sun J Du Q Zhang L Yang X Mechanical and dye adsorption properties of graphene oxide/chitosan composite fibers prepared by wet spinning Carbohydrate Polymers 2014 102 755 761 10.1016/j.carbpol.2013.10.094 24507344

[61] Hu H Wang X Wang J Liu F Zhang M Microwave-assisted covalent modification of graphene nanosheets with chitosan and its electro rheological characteristics Applied Surface Science 2011 257 7 2637 2642 10.1016/j.apsusc.2010.10.035

[62] Mahdavinia GR Marandi GB Pourjavadi A Kiani G Semi-IPN carrageenan-based nanocomposite hydrogels: synthesis and swelling behavior Journal of applied polymer science 2010 118 5 2989 2997 10.1002/app.32700

